# Cross-layer model design in wireless ad hoc networks for the Internet of Things

**DOI:** 10.1371/journal.pone.0196818

**Published:** 2018-05-07

**Authors:** Xin Yang, Ling Wang, Jian Xie, Zhaolin Zhang

**Affiliations:** School of Electronic and Information, Northwestern Polytechnical University, Xi’an, Shaanxi, China; Hong Kong Polytechnic University, HONG KONG

## Abstract

Wireless ad hoc networks can experience extreme fluctuations in transmission traffic in the Internet of Things, which is widely used today. Currently, the most crucial issues requiring attention for wireless ad hoc networks are making the best use of low traffic periods, reducing congestion during high traffic periods, and improving transmission performance. To solve these problems, the present paper proposes a novel cross-layer transmission model based on decentralized coded caching in the physical layer and a content division multiplexing scheme in the media access control layer. Simulation results demonstrate that the proposed model effectively addresses these issues by substantially increasing the throughput and successful transmission rate compared to existing protocols without a negative influence on delay, particularly for large scale networks under conditions of highly contrasting high and low traffic periods.

## Introduction

Nowadays, the Internet of Things (IoT) has greatly changed the way we live, work and study [1]. As one of the most important and critical part of the IoT, considerable progress has been made in the field of wireless ad hoc networks (WANET) recently, particularly with respect to improving their transmission performance [2]. WANET are networks in which nodes can communicate with others by wireless transmissions without the help of centralized coordinator [3]. Nowadays, many efforts are made in the improvement of networks performance like throughput. But WANETs still suffer from unbalanced resources between high traffic and low traffic periods, which constrains their transmission performance.

In response to these ongoing issues, numerous researchers have focused on media access control (MAC) protocols and code caching schemes in the physical (PHY) layer for WANETs [4]. For caching technique, it is an essential way to enhance the throughput and reduce latency of wireless networks [5]. The key point of caching is duplicating contents in memories distributed across the network, which can then be exploited to deliver requested content. Almotairi and Shen [6] proposed a distributed multi-channel MAC protocol to improve the throughput of networks. However, the protocol demonstrated unstable performance when network traffic fluctuated between high and low levels. Jiang and Du [7] presented a prediction-based time division multiple access (TDMA) MAC protocol for wireless networks denoted as PTMAC. While the protocol reduced transmission collision effectively, the throughput of networks was not improved. Moreover, it is subjected to transmission directions. A novel network coding aware cooperative MAC protocol denoted as NCAC-MAC has been proposed [8] that improves the throughput and reduces the delay of wireless networks. However, the collision of networks limits further improvement. Regarding the various code caching schemes developed, an uncoded caching scheme [9] has demonstrated relatively poor caching efficiency, while centralized code caching has demonstrated an index coding problem. Compared to the centralized code caching scheme, decentralized code caching (DCC) is more flexible, and can adapt to different numbers of user nodes. In addition, the DCC scheme can alleviate transmission congestion more effectively than the centralized scheme.

This paper presents a fundamentally novel cross-layer model based on modifications in the MAC layer and PHY layer to address the low throughput problem and reduce the transmission congestion of WANETs, especially for burst transmission networks. Here, a new content-division multiplexing (ContDM) transmission scheme is applied in the MAC layer, and is assisted by DCC in the PHY layer. Collectively, the proposed model is denoted as the ContDM-DCC (CDDC) model. Use of the ContDM scheme in the CDCC model allows for the effective employment of the transmission content in networks. This scheme also promotes efficient transmission during high traffic periods and makes better use of channels during low traffic periods. Use of DCC in the CDCC model improves network flexibility and facilitates the implementation of the model in large scale WANETs. The combination of the ContDM scheme with DCC allows the proposed model to solve the unbalanced resource problem by utilizing the characteristics of high and low traffic in networks. The solution of this problem significantly improves the transmission performance of WANETs. Simulation results demonstrate that the proposed model can enhance the throughput and packet deliver rate (PDR) in WANET systems.

## Transmission model description

The overall transmission process includes two stages: placement stage and delivery stage. The placement stage is initiated when the WANET traffic is low, and the delivery stage occurs when network traffic is high. In this work, we assume that the content request distribution conforms to a Zipfian distribution, which can distinguish between content with high and low frequencies of occurrence [10]. To briefly describe the Zipfian distribution, we assume a number of elements *N*, which, in our model, is the number of data packets that must be transmitted at the same time. The rank of each element is denoted by *k* and *s* is the value of the exponent characterizing the distribution. Therefore, the frequency of elements of rank *k* is obtained as $f(k,s,N)=\frac{1/k^{s}}{\sum_{n=1}^{N}\left(1/n^{s}\right)}$.

We employ the Pareto distribution in the proposed model to distinguish between content with high and low frequencies of occurrence. The Pareto distribution is regarded as a continuous counterpart of the Zipfian distribution, and has a cumulative distribution function (CDF) given as *f*_*x*_(*x*) = 1−(*x*_*m*_/*x*)^*α*^ for some number *x* ≥ *x*_*m*_, where *x*_*m*_ is the minimum possible value of a random variable *X* whose values are consistent with a Pareto distribution, and *α* is a positive parameter denoted as the Pareto index. In this letter, we simplify the processing by selecting *α* = log_4_5 = log5/log4, which is approximately 1.161. This is also denoted as the 80–20 law, where the top 20% of the content with the highest frequency of occurrence accounts for 80% of the transmission tasks in a network. The 80–20 law is widely used in information theory and information transmission.

In the placement stage, nodes pre-fetch high-likelihood content from the server, and save the content as cache in their memory. This represents the preparation phase of ContDM transmission. It is worth noting that the server in the proposed networks acts as a backend database. It does not control or affect the accessing of nodes. All nodes still make up an ad hoc network. The placement stage is regarded as the initialization and setting of the transmission. Here, high-likelihood content is directly sent by the nodes from their cache rather than through the server, which simplifies the transmission process. Meanwhile, low-likelihood content is still saved on the server, and sent by the server to the nodes when required. The simplified transmission process obtained by the caching of high-likelihood content can improve the transmission performance of WANETs effectively by avoiding traffic congestion in transmission channels among servers.

**Fig 1:**
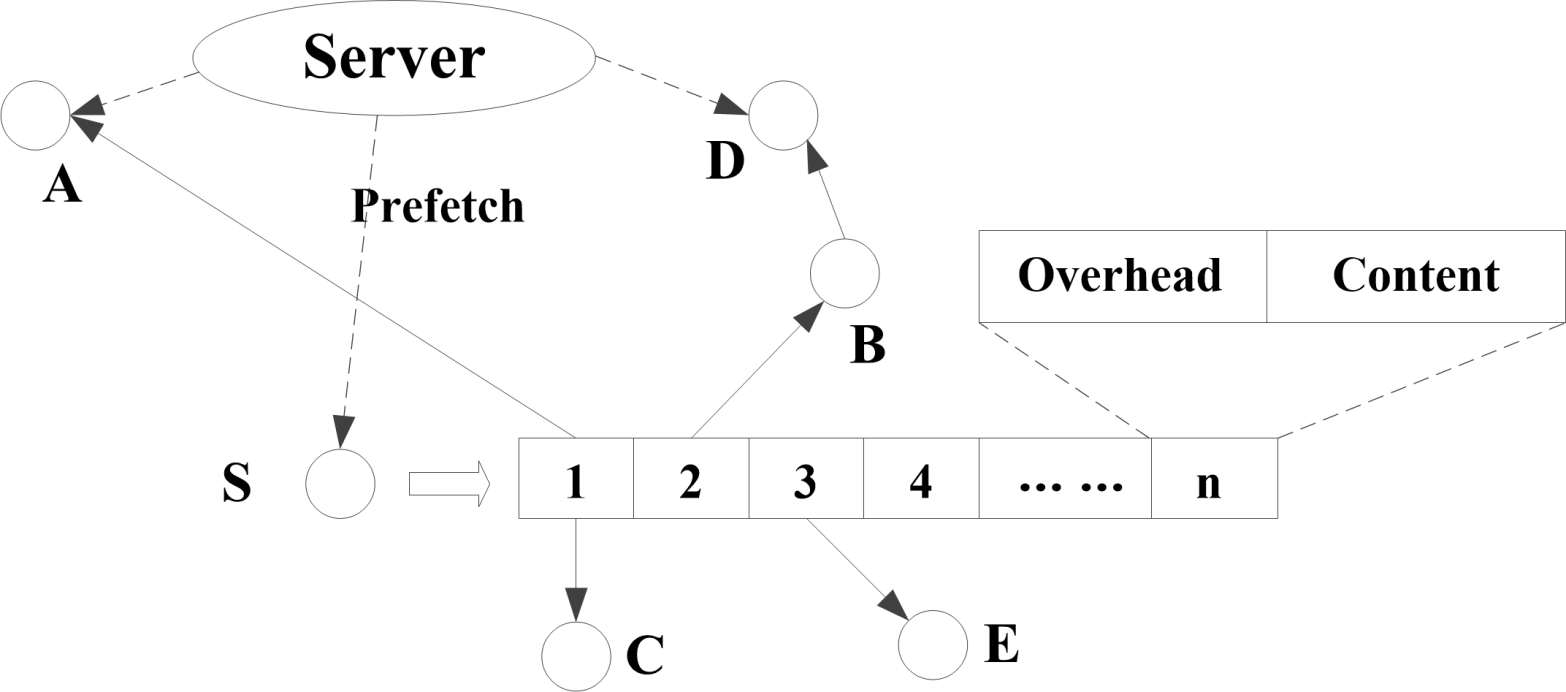
Content delivery process. In content delivery process of the proposed transmission model, we assume that the sender node S has pre-fetched and cached n content items from the server, some of which are subsequently sent to various receiving nodes A–E.

At the first step of the delivery stage, each user node presents requests for its required content. Then, neighboring nodes seek content in their cache and send useful content to the corresponding user nodes according to the ContDM scheme. The content in its neighbors are pre-fetched in the cache in low traffic period. In ContDM scheme, each neighboring node seeks to divide information into content items and to transmit content to corresponding receiving users, some of which require the same high-likelihood content. This process is illustrated in Fig 1, where node S is the sender, nodes A, B, C, and E are within the transmission range of S, node D is within the transmission range of B, but not within the transmission range of S. The cache of S contains *n* items of high-likelihood content based on the Zipfian distribution. Each content item has an overhead consisting of the location and size of the content. In Fig 1, S receives requests from neighboring nodes. According to the requests, S sends content 1 to A and C, sends content 2 to D with a hop via B, and sends content 3 to E. Node C only wants content 1, whereas A and D want low-likelihood content from the server. As a result of the pre-fetch operation involving S, the server can reduce its transmission pressure, and the performance of the network is thereby improved. Therefore, the ContDM scheme enhances WANET efficiency by conducting pre-fetching operations during low traffic periods, and the transmission performance of WANETs is improved during high traffic periods when an increasing number of users require high-likelihood content.

In the proposed model, we utilize DCC in the transmission process. In addition, DCC is introduced for WANET in which the data packets are encoded uniformly at random and cached. These characteristics can help to improve the throughput of the networks. The content is denoted according to the receiving node *N*, and every content item is divided into eight sub-contents:

$$\mathrm{Content}\;x(N)=(N_{\emptyset },N_{1},N_{2},N_{3},N_{12},N_{13},N_{23},N_{123}),$$ (1)

where *x* ∈ [1,2,3……*n*] represents the content items. For the example presented in Fig 1, content 1 requested by A is presented as

$$\mathrm{Content}\;1(A)=(A_{\emptyset },A_{1},A_{2},A_{3},A_{12},A_{13},A_{23},A_{123}),$$ (2)

which is the same as that requested by C. Content items 2 and 3 requested by nodes D (via B) and E, respectively, are divided similarly as content 1. We assume that, in this delivery stage, three content items (1, 2, and 3) are required for transmission. Firstly, S sends the coded packet *A*_23_ ⊕ *B*_13_ ⊕ *E*_12_, where ⊕ denotes bitwise XOR, because it is useful to all receivers. Then, S sends packets *A*_2_ ⊕ *B*_1_, *A*_3_ ⊕ *E*_1_, and *B*_3_ ⊕ *E*_2_ because they are useful to nodes A and B, A and E, and B and E, respectively. Finally, S sends *A*_∅_, *B*_∅_, and *E*_∅_, which are useful to nodes A, B, and E, respectively. Clearly, the content required by D is the same as that required by B, and that of C is the same as A. The server utilizes the DCC scheme when sending content to A and D as well. Therefore, DCC addresses the problem of asynchronous users, and improves the flexibility of networks.

DCC can also improve transmission performance in multi-hop transmission. To illustrate this advantage of DCC, the multi-hop transmission process of the proposed model is presented in detail based on the example shown in Fig 1, which describes the transmission route from S to D via B. According to the definition given in (1), we divide the content requested by D into eight sub-contents:

$$\mathrm{Content}\;2(D)=(D_{\emptyset },D_{1},D_{2},D_{3},D_{12},D_{13},D_{23},D_{123}).$$ (3)

**Fig 2:**
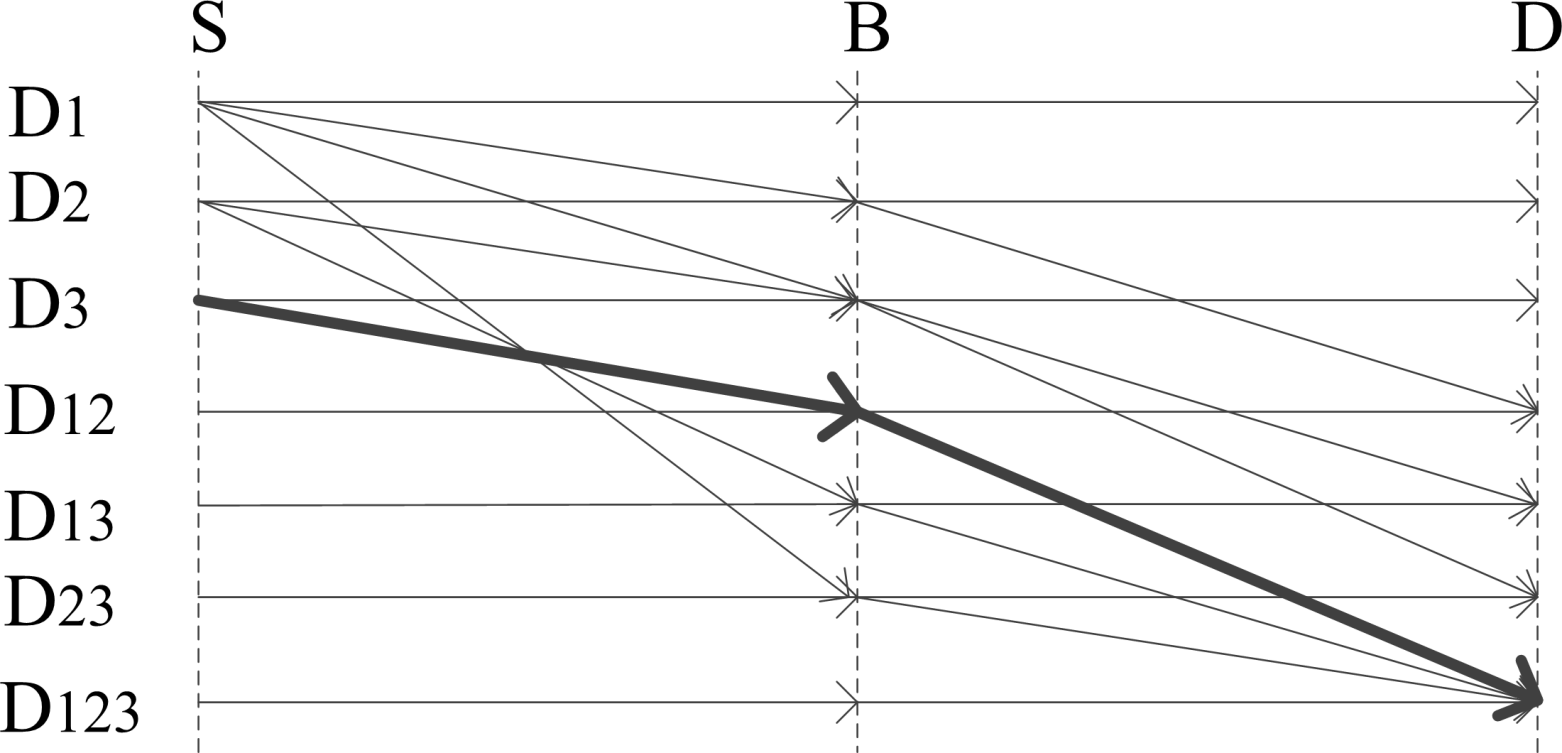
Transmission example. It is an example based on Fig 1 of two-hop transmission from node S to node D via node B as a relay node based on content structured according to DCC.

In this way, B acts as a relay node in the transmission. This DCC process is illustrated in Fig 2, where the straight lines with arrows represent transmission routes and directions from S to B and B to D. Hence, as shown by the bold line in Fig 2, if D requires *D*_123_, DCC takes advantage of the fact that B has already received and cached *D*_12_ prior to this transmission, and S need only send *D*_3_ to B, whereupon B sends *D*_123_ to D to complete the transmission. In this way, transmission time and collisions are reduced, resulting in increased throughput.

**Fig 3:**
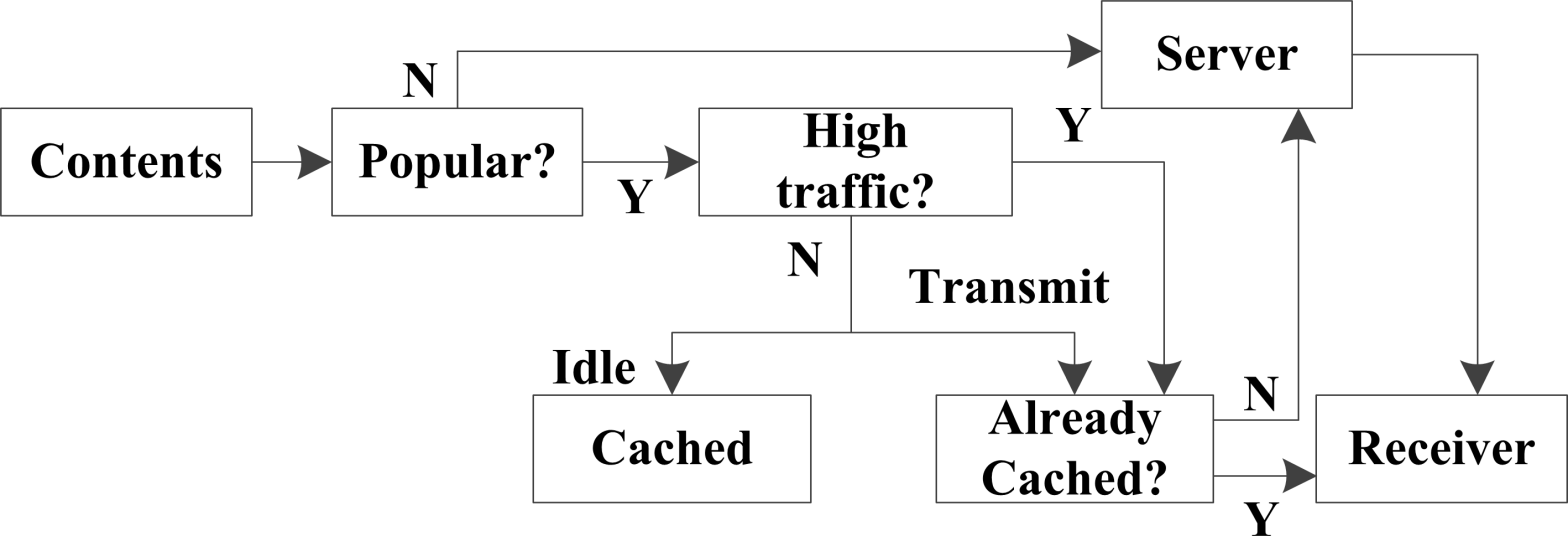
Algorithm flow chart of the proposed transmission model. The algorithm is based on Yes/No (Y/N) judge model.

The process flow of the proposed model is illustrated in Fig 3. As discussed, high-likelihood content is determined according to the 80–20 law, and high-likelihood content is cached by nodes during the idle time of low traffic periods, preparing for transmission to receivers. If the high-likelihood content required by a receiver has not been pre-fetched and cached, its request will be sent by the server. All content of low likelihood is transmitted by the server in the WANET system.

## Analysis and simulations

### Analysis

We first present a performance analysis of the proposed model. In the proposed network, we employ a retransmission scheme based on the carrier-sense multiple access with collision avoidance (CSMA/CA) mechanism, such that, if a sender node *i* fails in contending for transmission or the packets are not successfully received by a receiver node, node *i* will retransmit the packet after a back-off period in the range [0,*w*_*i*_), where $w_{i}=2^{\omega_{i}}$, and the value of *ω*_*i*_ depends on the number of unsuccessful transmission packets sent by i. In our system, the total number of nodes participating in transmission is n, and the set of these nodes is represented as *S*_*n*_. The set *S*_*c*_ represents the set of nodes contending for transmission. If a node in *S*_*c*_ has a transmission task, it will send packets within a period $\underset{i\in S_{C}}{\max}(w_{i})-1$. Otherwise, the transmission is failed. Thus, the probability of a packet being successfully transmitted for *S*_*c*_ can be given by

$$P_{SC}=1\text{-}P[\underset{i\in S_{C}}{\max}(w_{i})-1],$$ (4)

where $P[\underset{i\in S_{C}}{\max}(w_{i})-1]$ represents the probability that packet transmission has failed. Therefore, the probability of successful transmission, herein defined as the *PDR*, can be expressed as

$$P_{S}=P_{SD}+(1-P_{SD})\sum_{S_{c}\subseteq S_{n}}\prod_{i\in S_{c}}P_{SR_{i}}\prod_{j\in (S_{n}-S_{c})}(1-P_{SR_{j}})P_{SC},$$ (5)

where *P*_*SD*_ represents the probability of successful transmission without retransmission, $P_{SR_{i}}$ and $P_{SR_{j}}$ represent the probability of successful retransmission for nodes *i* and *j*, respectively, and *P*_*C*_ represents the probability of a node contending for transmission. In addition, we assume that the content requiring transmission is a Markov process. According to the example shown in Fig 2, the probability that content *D*_*a*(*n*+1)_ must be transmitted at time *T*_*n*+1_ is given as follows.

$$P(T_{n+1}=D_{a(n+1)}|T_{1}=D_{a1},T_{2}=D_{a2}\ldots T_{n}=D_{an})=P(T_{n+1}=D_{a(n+1)}|T_{n}=D_{an})$$ (6)

Here, *D*_*a*1_,*D*_*a*2_…*D*_*a*(*n*+1)_ ∈ Content 2(D).

The throughput of the presented model is defined as

$$Th=\frac{L_{S}(\mathrm{length}\;\mathrm{of}\;\mathrm{successful}\;\mathrm{transmission})}{T_{S}(\mathrm{time}\;\mathrm{of}\;\mathrm{successful}\;\mathrm{transmission})}=\frac{\sum_{i=1}^{S_{n}}P_{S}P_{n_{i}}}{\sum_{i=1}^{S_{n}}t_{n_{i}}P_{n_{i}}}.$$ (7)

Here, $P_{n_{i}}$ is the stationary probability of the Markov process discussed above, and $t_{n_{i}}$ is the total transmission time of node *i*, which can be calculated as

$$t_{n_{i}}=t_{s_{i}}+t_{e_{i}}+t_{c_{i}},$$ (8)

where $t_{s_{i}}$, $t_{e_{i}}$, and $t_{c_{i}}$ are the successful transmission time, empty slot time, and contending time of node *i*, respectively.

### Simulations

In the simulations, we assume that the collection of all nodes in the WANET covers an area 2000 m × 2000 m square, and that the transmission range of each node is 20 m. The simulations were conducted using the NS-3.26 discrete event network simulator. The parameters are summarized in Table 1. We compared the throughput and *PDR* performances of our CDCC model with those of the uncoded caching model, centralized code caching model, PTMAC, and NCAC-MAC. High-likelihood content was determined according to the 80–20 law in the simulations, and the CDCC model was simulated with *w* = 2^3^ = 8 or *w* = 2^4^ = 16. In addition, PTMAC does not use back-off scheme to reduce collision, NCAC-MAC utilizes random back-off scheme, and uncoded caching and centralized coded caching are not depend on back-off period. Hence, we just define the values of back-off period for CDDC to show the difference of performance.

**Table 1:** Parameters employed in the simulations.

Parameters	Values
Data packet	1024 bits
ACK	64 bits
RTS	64 bits
CTS	64 bits
SIFS	28×10^−6^ s
DIFS	128×10^−6^ s
Slot time	50×10^−6^ s

**Fig 4:**
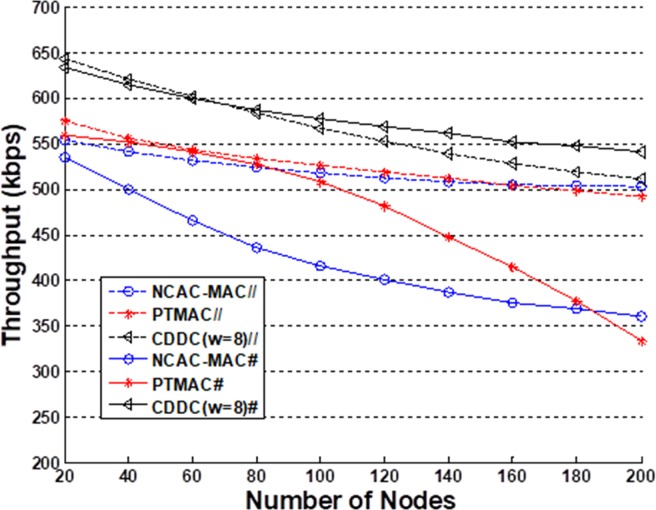
Throughput comparison with the other models. The simulation is based on two conditions, 25%–75% condition and 10%–90% condition, respectively.

**Fig 5:**
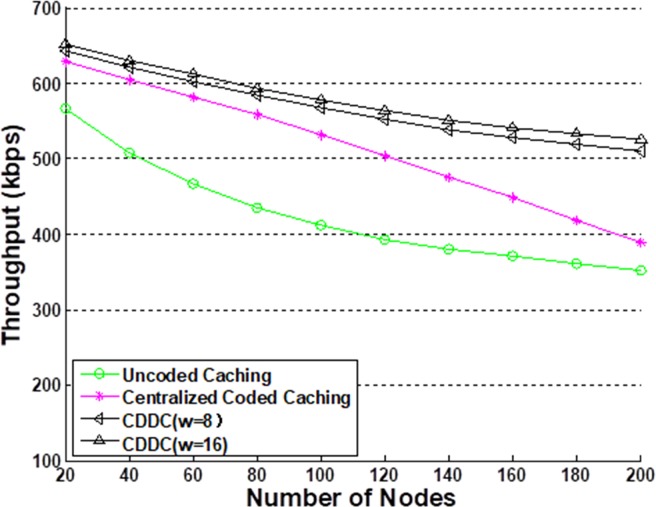
Throughput comparison with the other models. This comparison is based on 25%–75% condition.

**Fig 6:**
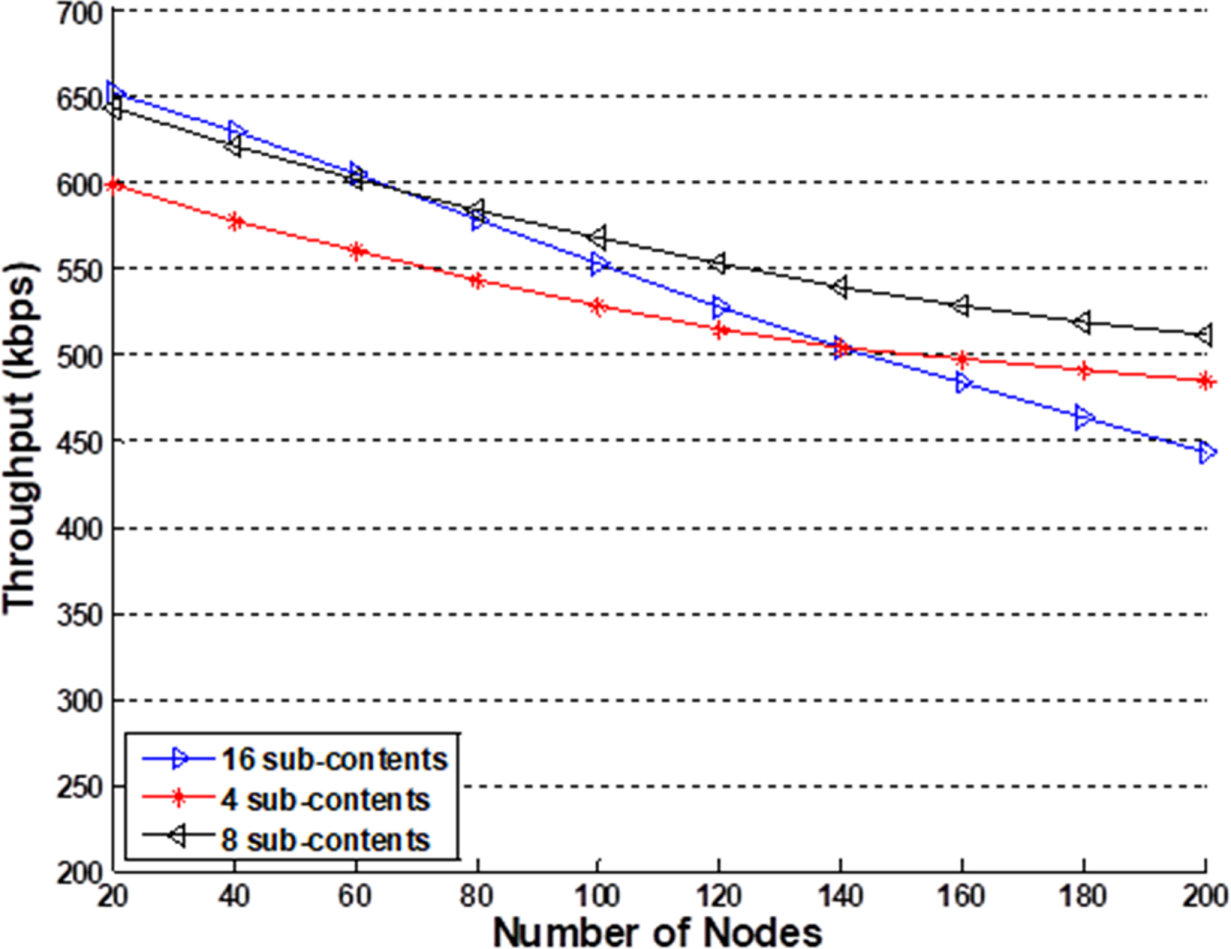
Throughput of different sub-contents. This simulation is based on 25%–75% condition.

Fig 4 shows the simulated throughput versus the number of nodes for the various transmission models considered. To include obvious high and low traffic periods in the simulations, we divided the simulation period into 2*N* equal periods, and assigned *M* slots to each period, where *N* and *M* are integers. Low traffic periods were assigned to the 1st, 3rd, …, (2*N* − 1)th periods, where an average of either 25% or 10% of the nodes of all slots had transmission tasks, while high traffic periods were assigned to all other periods, where an average of either 75% or 90% of the nodes of all slots had transmission tasks. These are respectively represented herein as the 25%–75% condition (denoted in the figures by '//') and the 10%–90% condition (denoted in the figures by '#'). We employed *N* = 5 in the simulations. The simulation results shown in Fig 5 and Fig 6 employ only the 25%–75% condition.

From Fig 4, we note that, under the 25%–75% condition, the CDCC model with *w* = 2^3^ = 8 has a throughput that is nearly 12% and 18% greater than those of PTMAC and NCAC-MAC, respectively, when the number of nodes is less than 140. The main reason for this greater throughput is that coded caching helps the ContDM scheme to utilize high-likelihood content efficiently. In addition, fewer nodes lead to a faster coded caching process. For greater than 160 nodes, NCAC-MAC has a better performance than PTMAC owing to its cooperative scheme, but still has a slightly smaller throughput than that of the CDCC model. The reason is that the greater number of nodes in the CDCC model can provide more high-likelihood content, which can reduce transmission pressure and improve throughput. Under the 10%–90% condition, we note that the CDCC model demonstrates a significantly higher throughput than the other models considered. The reason for this much higher throughput is that the more highly contrasting high and low traffic periods allow for nearly all high-likelihood content to be cached in low traffic periods, which substantially reduces transmission pressure on servers during high traffic periods. The throughput of PTMAC obviously decreases with an increasing number of nodes because the collision avoidance scheme is influenced by transmission from all directions during high traffic periods. In addition, NCAC-MAC cannot adapt to the fluctuating traffic, and demonstrates 20% to 30% less throughput than the CDCC model. Fig 5 shows that the proposed CDCC scheme provides higher throughput than the uncoded caching and centralized code caching models under the 25%–75% condition. For the condition of 50%-50% high-low traffic, the proposed protocol shows a similar throughput to NCAC-MAC when the number of nodes is less than 100 and a less throughput when there are more than 100 nodes in the networks. It means that CDDC is a better choice in burst transmission networks. In addition, we note that a larger value of *w* also provides a higher throughput.

Fig 5. shows the reason why we select 8 sub-contents scheme in the simulations. In the simulations, the high-low-traffic rate is denoted as 25%-75%. The 4 sub-contents scheme, as described in (9), does not take full use of the sub-contents scheme, so when the number of nodes is small, the throughput is unsatisfactory. On the other hand, the 16 sub-contents scheme, as described in (10), works worse in large scale nodes networks. Because too many contents transmitted by large numbers of nodes more easily lead to collisions in the transmission. That is why the throughput of 16 sub-contents decreases significantly when the number of nodes is more than 100.

$$\mathrm{Content}\;x(N)=(N_{\emptyset },N_{1},N_{2},N_{12}),$$ (9)

$$\mathrm{Content}\;x(N)=(N_{\emptyset },N_{1},N_{2},N_{3},N_{4},N_{12},N_{13},N_{14},N_{23},N_{24},N_{34},N_{123},N_{124},N_{134},N_{234},N_{1234}).$$ (10)

**Fig 7:**
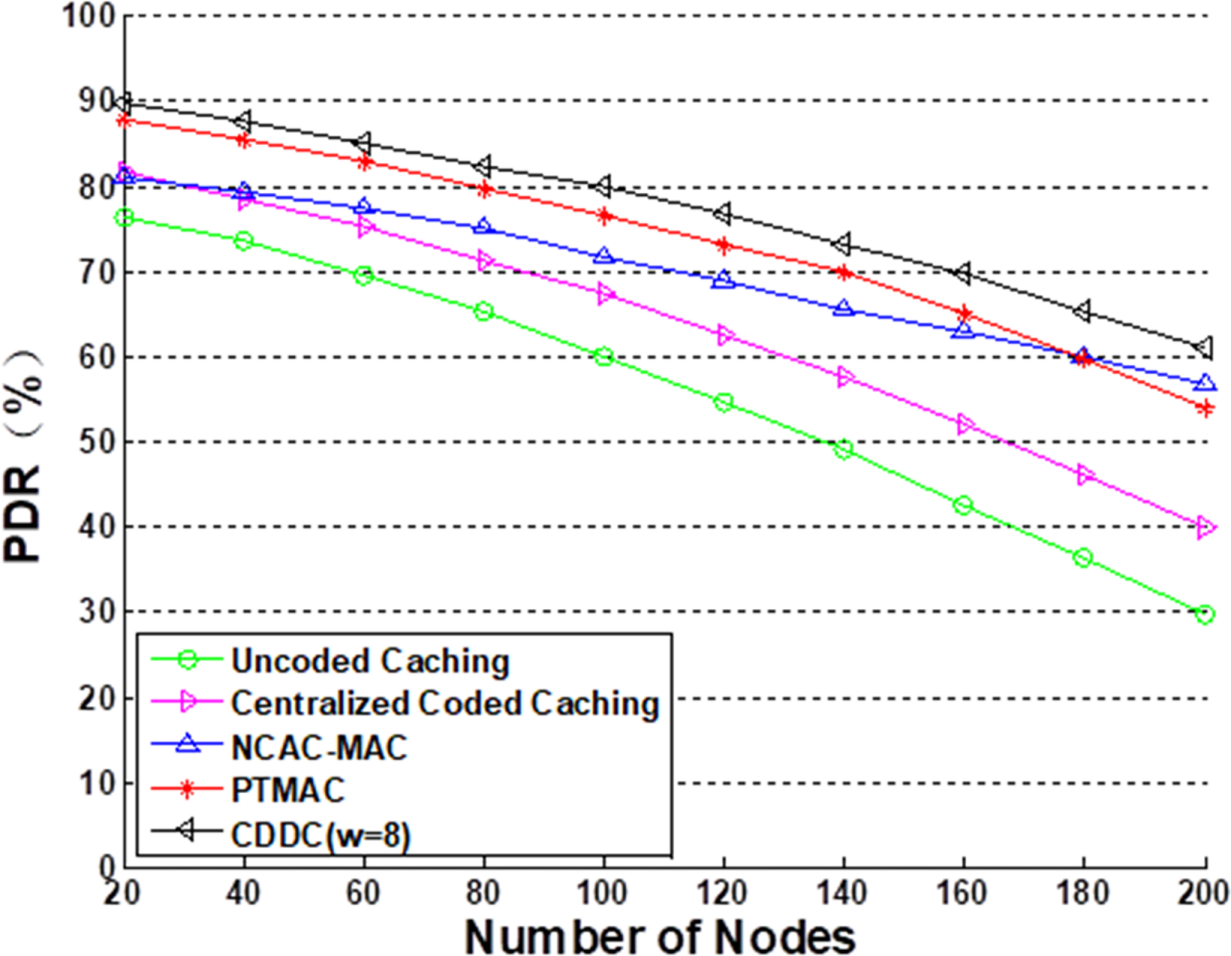
PDR values of the four transmission models. This simulation is based on 25%–75% condition.

Fig 7 shows the *PDR* values of the five models under the 25%–75% condition, where the CDCC model was simulated with *w* = 2^3^ = 8. *PDR* is a crucial evaluation of network performance, and also influences the throughput of networks. We note that the *PDR* values of all five models decrease with an increasing number of nodes. The CDCC model demonstrates the highest *PDR* among the four models considered due to its use of DCC and the effect of caching on the retransmission scheme. Here, if content is unsuccessfully transmitted, the sender only needs to retransmit the missing sub-content rather than the entire content. While the *PDR* values of both PTMAC and NCAC-MAC are only 3–5% less than that of the CDCC model, the *PDR* values of the other two models considered are as much as 30% less than that of CDCC. The reasons for these substantially lower performances is that uncoded caching disturbs the asynchronous demand of networks and leads to a greater number of transmission collisions than CDCC, and centralized coded caching cannot handle with ContDM scheme efficiently in the network.

**Fig 8:**
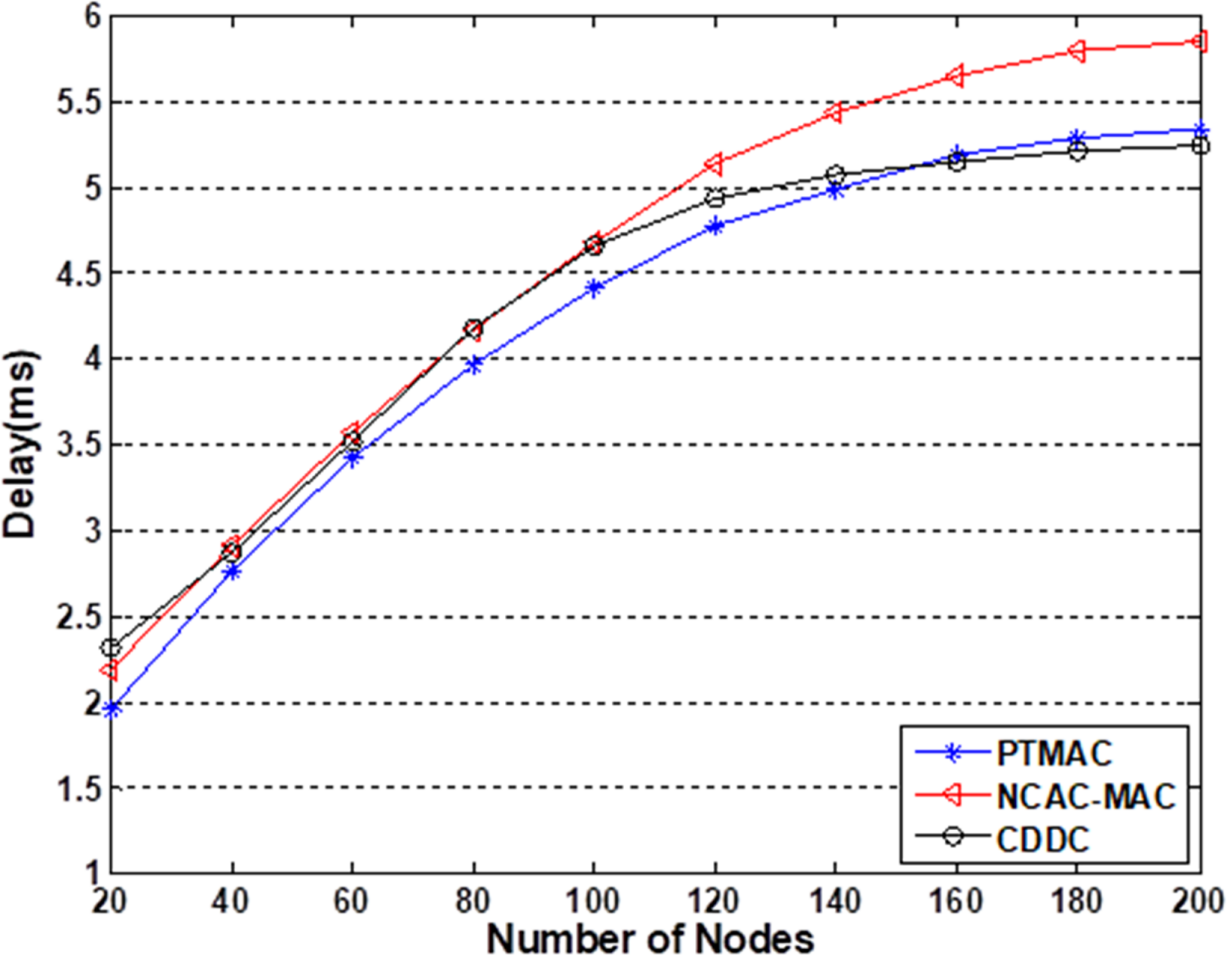
Delay of the three transmission models. This simulation is based on 25%–75% condition.

As shown in Fig 8, we simulate the delay of CDDC and compare to PTMAC and NCAC-MAC. It is obvious that PTMAC performs better when the number of nodes is small than 140. CDDC has a similar delay to NCAC-MAC when the number of nodes is less than 100. However, for a large scale of networks with more than 160 nodes, CDDC has an about 3% and 20% delay than PTMAC and NCAC-MAC. The reason is that more nodes provide more popular contents caching, in this way, nodes can obtain contents more easily and reduce the traffic load and delay. Meanwhile, when the number of nodes is less than 100, the advantage of popular contents caching cannot counteract the delay which is caused by computational work, so CDDC does not have a better performance on delay reducing than others.

## Conclusion

In this paper, we present a cross-layer transmission model for WANETs based on ContDM in the MAC layer combined with DCC in the PHY layer. In the proposed model, the ContDM scheme can improve the transmission performance of a WANET by making full use of low traffic periods to reduce transmission pressure during high traffic periods. Relative to the uncoded caching and centralized code caching schemes, DCC is more flexible, making it well suited for use in large scale WANETs. In addition, DCC can also reduce transmission congestion. Simulation results show the improvements on the transmission performance. These improvements are also of great benefit to practical WANET applications such as the IoT and vehicle networks, particularly for burst communication systems. In the future, we will focus on reducing energy consumption and developing applications of the proposed transmission model.
